# Recreational Physical Activity Ameliorates Some of the Negative Impact of Major Depression on Health-Related Quality of Life

**DOI:** 10.3389/fpsyt.2013.00022

**Published:** 2013-04-02

**Authors:** Scott B. Patten, Jeanne V. A. Williams, Dina H. Lavorato, Andrew G. M. Bulloch

**Affiliations:** ^1^Department of Community Health Sciences, University of CalgaryCalgary, AB, Canada; ^2^Department of Psychiatry, University of CalgaryCalgary, AB, Canada; ^3^Mathison Center for Research and Education in Mental Health, Hotchkiss Brain Institute, University of CalgaryCalgary, AB, Canada

**Keywords:** depressive disorders, quality of life, physical activity, recreation, epidemiologic studies, longitudinal studies

## Abstract

**Background:** Major depressive episodes have a negative effect on health-related quality of life (HRQoL). The objective of this study was to determine whether recreational physical activity can ameliorate some of this negative impact.

**Methods:** The data source for the study was the Canadian National Population Health Survey (NPHS). The NPHS is a longitudinal study that has collected data from a representative cohort of 15,254 community residents. Sixteen years of follow-up data are available. The NPHS included: an instrument to assess MDE (the Composite International Diagnostic Interview Short Form for Major Depression), an inventory of recreational activities (each associated with hours of participation and estimated metabolic expenditures), and a HRQoL instrument (the Health Utility Index, Mark 3, or HUI3). Proportional hazard and linear regression models were used in this study to determine whether MDE-related declines in HRQoL were lessened by participation in an active recreational lifestyle.

**Results:** Consistent with expectation, major depression was associated with a significant decline in HRQoL over time. While no statistical interactions were observed, the risk of diminished HRQoL in association with MDE was reduced by physical activity. In a proportional hazards model, the hazard ratio for transition to poor HRQoL was 0.7 (95% CI: 0.6–0.8, *p* < 0.0001). In linear regression models, physical activity was significantly associated with more positive HRQoL (β = 0.019, 95% CI 0.004 to −0.034, *p* = 0.02).

**Conclusion:** Recreational physical activity appears to ameliorate some of the decline in HRQoL seen in association with MDE. Physical activity may be an effective tertiary preventive strategy for this condition.

## Introduction

Depressive disorders are among the most important contributors to disease burden in developed countries (World Health Organization, 2001; Wittchen et al., 2011). These disorders affect mortality (Wulsin et al., 1999; Lawrence et al., 2010; Patten et al., 2011), but their main impact is through diminished functioning and lower health-related quality of life (HRQoL). The most important depressive disorder, Major Depressive Disorder has an annual prevalence in North America of approximately 5% (Kessler et al., 2003; Patten et al., 2006). As these conditions are so common, effective strategies to reduce their impact will have a substantially positive effect on HRQoL at the population level. Physical activity is a candidate strategy.

It is not difficult to identify mechanisms by which physical activity may have a positive impact on outcomes of depressive disorders. Depressive disorders increase the risks of a variety of chronic physical conditions such as hypertension (Patten et al., 2009), diabetes (Brown et al., 2005), and heart disease (Gilmour, 2008). Physical activity may help to ameliorate these risks. Physical activity may also counteract negative dynamics that can perpetuate depression, such as the emergence of a lifestyle that is lacking in rewarding or enjoyable activity (Hopko et al., 2003). A growing literature has examined the role of exercise in treatment of depression. The clinical trial literature has been summarized in a recent Cochrane Review (Rimer et al., 2012). Only a few studies have examined quality of life as an outcome. Carta et al. reported that the physical subscale of the WHOQOL-Bref improved in a randomized trial among subjects receiving antidepressant treatment and adjunctive exercise, whereas this did not occur in a control group receiving only antidepressant treatment (Carta et al., 2008). Singh et al. also examined quality of life outcomes in a trial of high-intensity progressive resistance training in community dwelling adults >60 years old. Improvements were noted in several Medical Outcomes Study Short Form (SF-36) subscales, although only one of these, vitality, achieved statistical significance (Singh et al., 2005).

To our knowledge, no epidemiologic studies have examined the joint effects of physical activity and major depressive episode (MDE) on quality of life outcomes in major depression. The objective of this study was to examine these effects using a representative general population sample.

## Materials and Methods

The data source for this study was a Canadian prospective cohort study called the National Population Health Survey (NPHS) (Swain et al., 1999). This is a longitudinal study based on a nationally representative community sample assembled by Statistics Canada (Canada’s national statistical agency) in 1994/1995. Baseline interviews (mostly face to face) were carried out in 1994 and participants were re-interviewed every 2 years subsequently, usually by telephone. Statistics Canada reported a 69.7% rate of successful follow-up at completion of the project in 2010 (Statistics Canada, 2012).

The original NPHS longitudinal cohort included 17,276 participants in total, but the current analysis was restricted to 15,254 respondents who were over the age of 12 at the time of the initial 1994 interview. This subset was further restricted in specific analyses depending on the health transitions of interest to the study. For example, in the component of the analysis concerned with incidence of low HRQoL, those already having low HRQoL at the time of the baseline interview were excluded because they could not be considered at risk of developing this outcome.

The NPHS interview included the Composite International Diagnostic Interview Short Form (CIDI-SF) (Kessler et al., 1998) for Major Depression. This is a brief structured interview designed to identify people with a high probability of past year MDE. The CIDI-SF was developed using data from the National Comorbidity Survey in the US (Kessler et al., 1994), which used the DSM-III-R classification. The instrument consists of a modified subset of CIDI items and is scored using a predictive algorithm. For the current analysis, the 90% predictive cut-point was used. This scoring procedure requires endorsement of five symptom-based criteria (at least one of which must be depressed mood or loss of interest), providing face validity for the DSM-IV definition of MDE.

Each cycle of the NPHS also included items assessing participation in 21 recreational physical activities. Each activity was assigned a metabolic indicator (MET) value (Statistics Canada, 2004) representing an estimated metabolic energy cost (in kilocalories expended per kilogram of body weight per hour) which is expressed as a multiple of the resting metabolic rate. For example, the MET value for playing basketball is six, indicating that people playing basketball expend an estimated six times more energy per hour than people at rest. Daily estimated energy expenditure was then calculated from MET values based on the amount of time spent participating in each specified activity. A total estimated energy expenditure of 1.5 kcal/kg/day was used to categorize respondents into active or inactive categories. This level of activity corresponds approximately to 30 min of walking for exercise per day. The methodological approach to the assessment of leisure time physical activity was developed by the Canadian Fitness and Lifestyle Institute^1^.

Health-related quality of life was assessed in the NPHS using the Health Utility Index, Mark 3 (HUI3). The Health Utilities Index (HUI) is a system for measuring HRQoL and for producing preference-weighted health utilities. The HUI3 system was originally developed for the 1990 Ontario Health Survey (Horsman et al., 2003). The HUI covers eight attributes: vision, hearing, speech, ambulation, dexterity, emotion, cognition, and pain. Each level of each attribute is associated with an attribute-specific utility score with values ranging from 1.0 (the highest of the five or six options) to zero (the lowest). However, most commonly, the various health states are used to compute a multi-attribute score using a multiplicative multi-attribute algorithm (Feeny et al., 2002). The preference weights used in this algorithm derive from data collected in a survey employing standard gamble methods (Feeny et al., 2002). In the version used by Statistics Canada, perfect HRQoL is associated with an HUI3 score of 1.0, a state equivalent to death is assigned a score of zero and health states of less than zero are viewed as being worse than death. Additional information is available at the instrument’s website^2^.

Various questionnaires that provide sufficient information to describe health status have been developed for use with the HUI3. The version used by Statistics Canada refers to “usual” experience of various impairments (some other versions use past month or past week ratings). The instrument used by Statistics Canada in its national surveys is called the Comprehensive Health Status Measurement System (CHSMS). This instrument was included in the NPHS. Eight domains are covered by the CHSMS: vision, hearing, speech, mobility, dexterity, emotion, cognition. As noted above, each of the individual health states is assessed at several different levels, which leads to 972,000 possible unique health states, each of which is associated with a HRQoL value.

We were interested in examining associations between major depression, physical activity, and HRQoL from several different perspectives. A commonly employed interpretation of HUI3 data is a nominal one, with scores <0.70 being considered indicative of low HRQoL (Horsman et al., 2003). However, it is also of interest to examine uncategorized ratings, so we also treated the HUI3 ratings as a continuous variable in some analyses.

In preliminary descriptive and stratified analyses we confirmed that major depression was associated in the longitudinal data with declines in HRQoL. In order to evaluate effect modification by physical activity, proportional hazards models were used. Because the NPHS collected data at specific time points (every 2 years), grouped time models were used. These models were fit as generalized linear models of the binomial family with a complementary log–log link function. Jenkins (1997) outlines procedures for implementation of such analyses in STATA (Stata Corporation, 2005), the data analysis software used in all analyses reported here. The proportional hazards assumption was evaluated using a likelihood ratio test for time by exposure (major depression, physical activity) interactions.

We extended this analysis to examine changes in HUI3 scores during the NPHS follow-up without categorization. To accomplish this we calculated a change score by subtracting the 2010 HUI3 rating from the 1994 rating. These differences were found to be normally distributed, so we were able to use linear regression to model these changes in terms of MDE and physical activity during the intervening cycles.

The target population for the NPHS consisted of household residents. Residents of institutions, certain remote areas, Indian reserves and the Armed Forces were excluded from the sampling frame. The NPHS used a multi-stage sampling procedure that resulted in unequal selection probabilities and clustering. To correct for these design effects, Statistics Canada recommends a bootstrap procedure that uses a set of 500 replicate sampling weights. The NPHS sampling weights also include a non-response adjustment. Respondents who were lost to follow-up, died, or were institutionalized were censored in the analysis. This project was approved by the University of Calgary Ethics Review Board.

## Results

Table 1 presents a description of the study sample. Demographic characteristics of the full baseline sample are presented, but also for those below the cut-point of 0.7 and those at or exceeding this threshold. The Table shows that 13% of the sample already had low HRQoL at their baseline time point. This group was older, more likely to be female, more likely to be divorced, widowed, or separated and more likely to have low education. They were also more likely to be depressed and more likely to be physically inactive.

**Table 1:** **Demographic features of study sample (NPHS) at baseline (1994)**.

		NPHS; *N* = 15,254 [% (95% CI)]	HUI < 0.7; *N* = 2,250* [% (95% CI)]	HUI ≥ 0.7; *N* = 12,398* [% (95% CI)]
Gender	Male	49.2 (49.1–49.2)	42.9 (40.4–45.4)	49.4 (49.0–49.9)
	Female	50.8 (50.8–50.9)	57.1 (54.6–59.6)	50.6 (50.1–51.0)
Age (mean)		40.9 (40.8–41.0)	49.7 (48.7–50.7)	39.5 (39.2–39.7)
Marital status	Married/common law	59.0 (58.2–59.7)	54.6 (51.8–57.4)	59.3 (58.4–60.2)
	Single	28.9 (28.3–29.6)	23.0 (20.6–25.4)	30.1 (29.4–30.9)
	Widowed/separated/divorced	12.1 (11.6–12.6)	22.4 (20.3–24.4)	10.6 (10.0–11.1)
Education	Less than secondary or secondary school graduation	48.2 (47.2–49.3)	57.5 (54.7–60.3)	46.7 (45.6–47.8)
	Some post-secondary or post-secondary graduation	51.8 (50.7–52.8)	42.5 (39.7–45.3)	53.3 (52.2–54.4)
Depressed	Yes	5.6 (5.1–9.1)	14.1 (12.1–16.0)	4.3 (3.8–4.7)
	No	94.4 (93.9–94.9)	85.9 (84.0–87.9)	95.7 (95.3–96.2)
Physically active	Yes	41.6 (40.5–42.7)	32.7 (29.8–35.5)	43.1 (41.8–44.3)
	No	58.4 (57.3–59.5)	67.3 (64.5–70.2)	56.9 (55.7–58.2)

*The weighted proportion in the low HRQoL group at baseline was 13.4%, with 86.6% in the remaining “at risk” sample.

We initially focused on the 12,398 respondents that did not have low HRQoL at baseline, and were therefore at risk of making this transition during follow-up (see the right-hand column in Table 1). We modeled their risk of developing low HRQoL during follow-up based on their MDE and physical activity status, each of which were allowed to vary with time during follow-up. Among those who were active, there was a diminished risk of transition into the low HRQoL group during NPHS follow-up (HR = 0.7, 95% CI: 0.6–0.8). Those with MDE had an elevated risk of this transition (HR = 2.3, 95% CI: 1.8–2.8). When both variables were included in a single model along with an interaction term, the HR associated with that interaction term was 1.1 (95% CI 0.7–1.8), which was non-significant according to a Wald test (*p* = 0.57). As this is a multiplicative model, the lack of an interaction suggests that physical inactivity and major depression have a multiplicative relationship – recreational physical activity diminishes the risk of transition to low HRQoL to about 70% of what it would have been in view of the strongly negative effects of MDE.

Another way to examine these results is to code the physical activity variable such that it represents physical inactivity rather than activity. Coded this way the hazard ratio for physical inactivity was 1.4 (95% CI 1.3–1.6), indicating that inactivity increases the risk of transition to low QoL by about 40%.

Additional covariates were explored in the modeling, including: age; sex; divorced, widowed, or separated marital status; and low (less than secondary level) educational attainment. These variables (except sex) all predicted low HRQoL during follow-up, but none of them confounded the opposing associations of major depression and physical activity with low HRQoL. A model that included each of these variables simultaneously along with a major depression by physical inactivity interaction term resulted in a non-significant interaction (HR = 1.1, 95% CI 0.7–1.8), *p* = 0.60. This model, with removal of the interaction term, is presented in Table 2. The adjusted HR for physical activity was nearly unchanged in this analysis (HR = 0.8, 95% CI 0.7–0.9).

**Table 2:** **Proportional hazards model predicting HRQoL < 0.7 in the NPHS**.

Variable		HR (95% CI)	*p*-Value
Physical activity		0.8 (0.7–0.9)	*p* < 0.001
Major depression		2.9 (2.3–3.7)	*p* < 0.001
Female sex		1.1 (1.0–1.2)	*p* = 0.25
Age*		1.0 (1.0–1.0)	*p* < 0.001
Marital status	Single	1.4 (1.2–1.6)	*p* < 0.001
	Divorced, widowed, separated	1.3 (1.1–1. 4)	*p* = 0.001
Education < secondary level		1.4 (1.3–1.6)	*p* < 0.001

*Age was treated as a continuous variable in this analysis. The unrounded HR was 1.03, indicating a 3% increase in risk of the transition to low HRQoL with each increasing year of age.

As explained above, we also explored HUI3 as an uncategorized variable by examining the difference between baseline and end-point ratings. In this analysis, a slight negative change of 1.5% (α = −0.015, 95% CI −0.02 to −0.01, *p* < 0.0001) was observed in respondents without MDE, apparently representing a slight age-related decline over the 16 years of follow-up. There was a significantly greater decline in those with MDE: 4% (β = −0.025, 95% CI −0.05 to −0.002, *p* = 0.028). Variables also associated with more rapid decline in HRQoL were explored by including them initially one at a time in a series of linear regression models. Female sex was associated with a more rapid decline in HRQoL (β = −0.017, 95% CI −0.02 to −0.004, *p* = 0.008). Age at baseline, which was treated as a continuous variable, was also associated with diminishing HRQoL (β = −0.002, 95% CI −0.003 to −0.002, *p* < 0.0001). Educational status and marital status were not significantly associated with change in HRQoL.

In a separate analysis we identified respondents that were persistently physically active during follow-up (rather than treating this as a time-varying characteristic) and evaluated interactions between physical activity and MDE in another linear regression model. As with the previous analysis, a cross-product interaction term for depression and physical activity was not significant (β = −0.014, 95% CI −0.072 to 0.043, *p* = 0.62). When this variable was removed from the model the effect of MDE remained negative (β = −0.024, 95% CI −0.046 to −0.011, *p* = 0.04) and physical activity became associated with a significantly positive impact on HRQoL (β = 0.019, 95% CI 0.004 to −0.034, *p* = 0.02).

**Figure 1:**
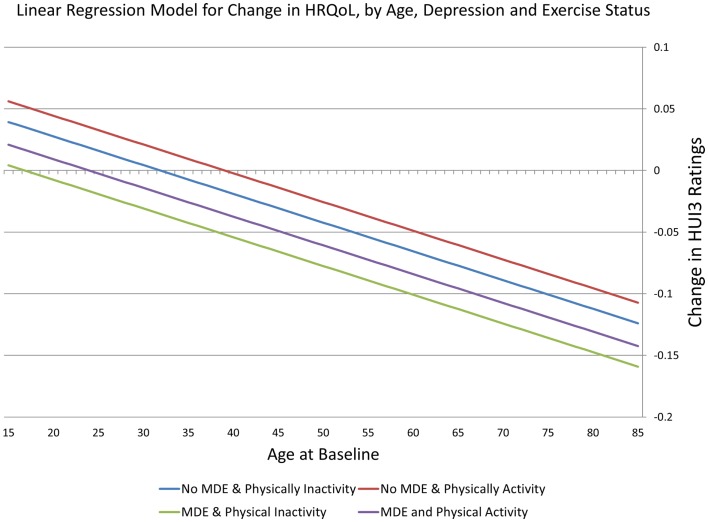
**Linear regression model for change in HRQoL**.

In a model containing the covariates listed in Table 1, the interaction term remained non-significant, as did marital status, sex, and education. Fitted values from a linear regression model including MDE, physical activity, and age are presented in Figure 1. The parallel nature of the regression lines reflects the removal of the non-significant interaction term from the model. Changes in HRQoL are positive in the youngest ages and then become negative at older ages. MDE is association with more negative changes, whereas this is partially offset by persistent physical activity.

In a final model, we categorized physical activity at two levels, sometimes physically active (i.e., in one or more of the 1–5 intervening NPHS cycles) or persistently physically active. After adjustment for age, which was again the only significant predictor of HRQoL changes, being sometimes physically active was associated with a small and non-significant positive effect (β = 0.011, 95% CI −0.009 to 0.033, *p* = 0.27), whereas the effect of persistent physical activity remained significant in this analysis (β = 0.027, 95% CI 0.003–0.050, *p* = 0.03).

## Discussion

There has been much discussion in the literature about the role of physical activity as a treatment or clinical management strategy for depression. A recent Cochrane review concluded that the evidence from high quality studies is generally positive, but that the effect on depression was small (Rimer et al., 2012). A possibility that has received much less attention is the possibility that participation in physically active recreational activities may lead to better quality of life outcomes. The epidemiologic data presented here suggests that it does.

There are several mechanisms that may explain this association. When people become depressed they often diminish their participation in recreational activities due to the anhedonia and fatigue that often accompanies depressive disorders. This may lead to lifestyle changes that become habitual and do not recover after the episode. On the other hand, people that maintain their participation in physical activity may derive benefits from this. Many other mechanisms are possible. For example, chronic disease incidence is elevated in MDE, and as chronic conditions are likely to contribute to declining HRQoL, physical activity may protect against declining HRQoL by diminishing chronic disease incidence. It should be acknowledged, however, that once a medical condition emerges participation in physical activity may be affected by that condition. Whereas it may appear that HRQoL declined to a greater extent as a result of MDE combined with physical inactivity, in reality the physical inactivity may have resulted from some other factor (such as an emerging medical condition) having a subsequent effect on HRQoL.

One of the limitations of the epidemiological data source used in this is study is that the interviews were spaced 2 years apart. As a result, the exact timing of changes within NPHS cycles cannot be discerned. For this reason, temporal relationships between depressive episodes, physical activity, associated factors such as medical conditions, and HRQoL changes cannot be clarified with certainty. It is therefore prudent to regard the findings as being suggestive, but not confirmatory, of a causal effect. Randomized controlled trials provide an appropriate vehicle for confirming these results. Future trials of exercise in MDE should including HRQoL measures in their assessment of outcome.

This study has several additional limitations. One is that the epidemiologic data set employed in the analysis was a general health survey that did not include sophisticated assessments either of MDE or of physical fitness. Instead, there was a reliance on abbreviated survey instruments to measure these variables. A related limitation is that it was not possible to make detailed adjustments for confounding variables, except for some fairly basic demographic variables. If replicated, however, these results highlight the potential of physical activity to diminish some of the negative impact of depressive disorders. This is potentially a valuable avenue for reducing the burden of depressive disorders on population health.

## Conflict of Interest Statement

The authors declare that the research was conducted in the absence of any commercial or financial relationships that could be construed as a potential conflict of interest.

## Footnotes

^1^http://www.cflri.ca

^2^http://www.healthutilities.com/hui3.htm
